# Parental selection for growth and early-life low stocking density increase the female-to-male ratio in European sea bass

**DOI:** 10.1038/s41598-021-93116-9

**Published:** 2021-06-30

**Authors:** Benjamin Geffroy, Manuel Gesto, Fréderic Clota, Johan Aerts, Maria J. Darias, Marie-Odile Blanc, François Ruelle, François Allal, Marc Vandeputte

**Affiliations:** 1 MARBEC, Univ Montpellier, CNRS, Ifremer, IRD, Palavas-Les-Flots, France; 2 Techn Section for Aquaculture, DTU Aqua, Technical University of Denmark, Willemoesvej 2, 9850 Hirtshals, Denmark; 3 Université Paris-Saclay, INRAE, AgroParisTech, GABI, 78350 Jouy-en-Josas, France; 4 Stress Physiology Research Group, Faculty of Sciences, Ghent University, Ostend, Belgium

**Keywords:** Metabolism, Reproductive biology, Ecology, Molecular biology, Physiology, Zoology

## Abstract

In European sea bass (*Dicentrarchus labrax*), as in many other fish species, temperature is known to influence the sex of individuals, with more males produced at relatively high temperatures. It is however unclear to what extent growth or stress are involved in such a process, since temperature is known to influence both growth rate and cortisol production. Here, we designed an experiment aiming at reducing stress and affecting early growth rate. We exposed larvae and juveniles originating from both captive and wild parents to three different treatments: low stocking density, food supplemented with tryptophan and a control. Low stocking density and tryptophan treatment respectively increased and decreased early growth rate. Each treatment influenced the stress response depending on the developmental stage, although no clear pattern regarding the whole-body cortisol concentration was found. During sex differentiation, fish in the low-density treatment exhibited lower expression of *gr1*, *gr2, mr,* and *crf* in the hypothalamus when compared to the control group. Fish fed tryptophan displayed lower *crf* in the hypothalamus and higher level of serotonin in the telencephalon compared to controls. Overall, fish kept at low density produced significantly more females than both control and fish fed tryptophan. Parents that have been selected for growth for three generations also produced significantly more females than parents of wild origin. Our findings did not allow to detect a clear effect of stress at the group level and rather point out a key role of early sexually dimorphic growth rate in sex determination.

## Introduction

In various fish species, sex is environmentally determined^1^. Different environmental cues have been identified as factors that can act on sex determination, with the most widely studied being undoubtedly temperature. Since the first discovery of its effects on *Menidia menidia*^2^, temperature sex determination (TSD) has been detected in a plethora of fish species^3^ and is now depicted in the light of global warming^4^. However, other abiotic or biotic conditions, such as water acidity^5^ or fish density^6,7^, have been identified as major factors affecting the sex of fish. In this regard, it has been hypothesized that stress (likely through the action of cortisol) could be a cornerstone of environmental sex determination (ESD) in fish^6,8^. The stress response in fish begins when the hypothalamic-pituitary-interrenal (HPI) axis is stimulated to release corticotropin-releasing factor (CRF) by the hypothalamus, which is carried by the portal system to the pituitary where it stimulates the synthesis and secretion of pro-opiomelanocortin (POMC)-derived peptides involved in the mediation and regulation of the stress response, including the secretion of the adrenocorticotropic hormone (ACTH). ACTH then binds to the melanocortin 2 receptor (MC2R) to stimulate glucocorticoid secretion in the interrenal cells^9^. In fish, cortisol is the main glucocorticoid, which is a ligand for two glucocorticoid receptors, GR1 and GR2 and a mineralocorticoid receptor, MR^10^. The steroidogenic acute regulatory (STAR) protein regulates the rate-limiting step in cortisol biosynthesis^11^. It transfers hydrophobic cholesterol, the cortisol precursor, across the aqueous barrier between the outer and inner mitochondrial membrane, where cortisol is synthesized by side chain cleavage of the P450scc complex. Pregnenolone, the metabolic intermediate in the biosynthesis of cortisol, then undergoes a series of isomerisations and hydroxylations by several enzymes, including the 21-hydroxylase (CYP21), 11β-hydroxylase (HSD11B1), and Cytochrome P450 11β (CYP11b) that finally transform 11-deoxicortisol to cortisol. Cortisol in turn induces increased energy metabolism to cope with stress. Although the specific role of the isoform 2 of HSD11B enzyme in teleost fishes is not well known, it has been suggested to regulate the balance of active versus inactive cortisol (cortisone)^12^. Cortisone is then converted into tetrahydrocortisone by the 5β-reductase enzyme and excreted mainly through urine. Several key genes coding for the above-mentioned enzymes have been shown to be modulated during ESD or sex-change in fish, such as *cyp21a*^13^, *cyb11B*^14,15^ or *hsd11b2*^16^. Yet, and somehow surprisingly, the various studies conducted on phylogenetically distant fish species led to the conclusion that, when stress is involved in sex determination, it always triggers maleness^8,17^. This raises fascinating questions regarding the adaptive potential of being male in stressful conditions^17^.

In both aquaculture and experimental conditions, different strategies can help to mitigate stress and improve welfare. For instance, a relatively low fish density has been shown to decrease stress levels in zebrafish (*Danio rerio*)^7^ and Japanese eel (*Anguilla japonica*)^18^. Moreover, the supplementation of fish diets with certain compounds can also help mitigate stress, as observed, for example, with tryptophan, which is a precursor of serotonin and of the hormone melatonin^19^.

The European sea bass (*Dicentrarchus labrax*), a major species for marine fisheries and aquaculture in Europe^20^, has a polygenic sex determination system that is affected by temperature^21–23^, where genetic sex determination (GSD) interact with ESD. High temperatures (> 20 °C) during the larval stage promote male sex determination^24,25^, and long-term (until juvenile stage) exposure to relatively low temperature (i.e*.* 16 °C) also results in masculinization^26^. Various studies have shown that future females exhibit higher growth rate when compared to future males, starting very early in the development^27–29^. The fact that early exposure to elevated temperature triggers maleness in European sea bass could thus be viewed as a paradox since relatively high temperature also generally promote fish growth in aquaculture conditions (i.e. food is not limited)^30^. This is why testing the stress hypothesis is particularly relevant in this species.

**Figure 1:**
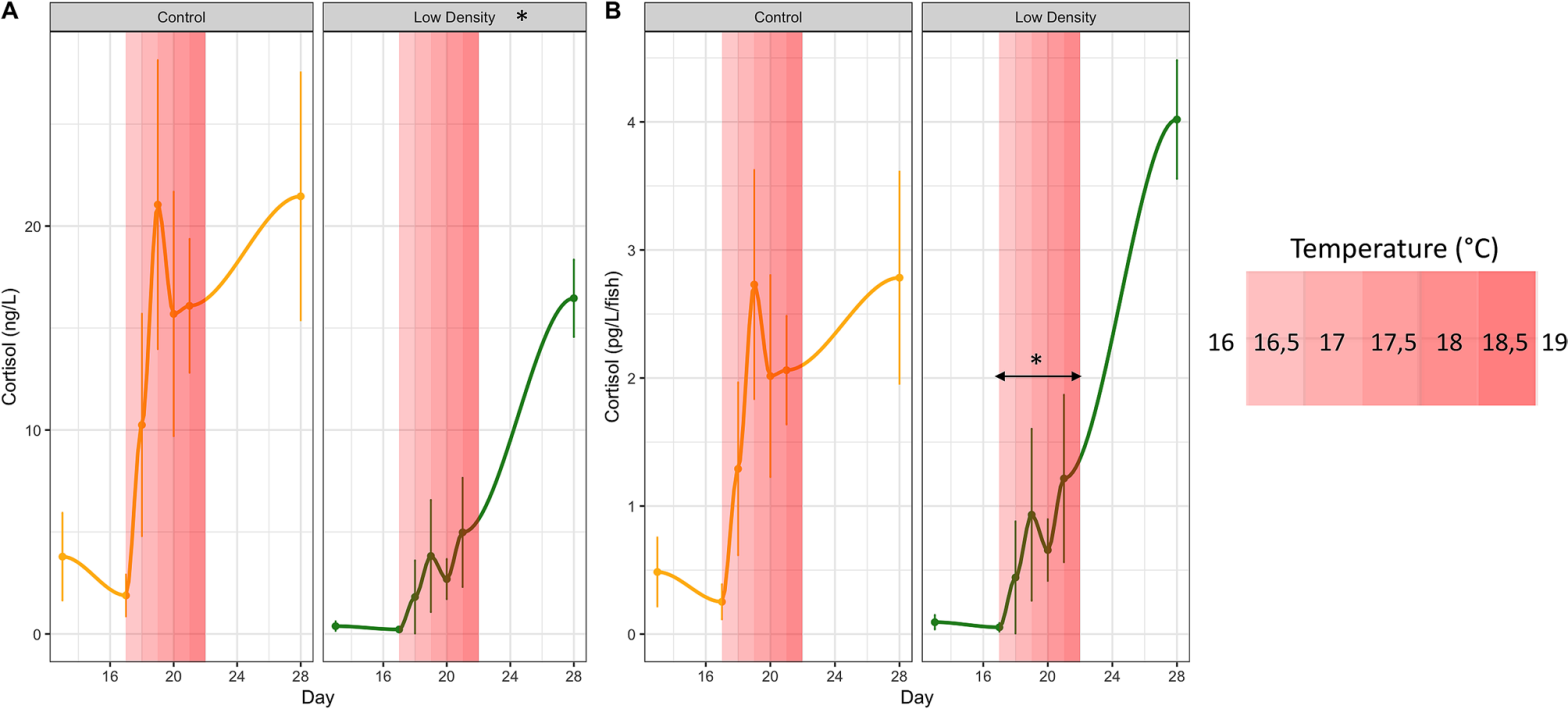
Cortisol production over time by European sea bass larvae reared at low or normal (Control) density and exposed to a gradual increase of temperature over 5 days. (**A**) Mean cortisol (ng/L) and (**B**) Mean cortisol produced per fish (pg/L/fish) represented by points (mean + SE), released into the water by developing larvae (from 13 to 28 dph) exposed to a gradual increase of temperature from 16 to 19 °C (0.5 °C per day from 17 dph, represented by red rectangles of increasing intensities). “*” indicates significant differences in cortisol release during the increases of temperature between the low-density treatment and the to control (p < 0.05).

The objective of the current study is to assess whether stress and/or growth might be mechanistically involved in the known effect temperature has on sex determination in sea bass, and assess how the genetic background of fish interacts with environment. Therefore, we designed two experimental conditions that were expected to reduce stress levels before and during the period of sex determination in European sea bass, namely dietary tryptophan and low stocking density. We also assessed the effect of genetic origin of parents (wild or selected for growth) on sex ratio. In order to mimic commercial sea bass hatchery conditions, we increased temperature from 16 to 19 °C between 16 and 21 days post hatching (dph), long before the supposed window of molecular sex determination (between 110 and 150 dph)^31,32^. We monitored cortisol release in the water during this period for all experimental conditions to evaluate to what extent temperature correlated with this stress hormone. Fish were then sampled during the course of the experiment at the “flexion”, “all fins” and “juveniles” stages, that correspond to key moments of the stress axis development^33,34^ and sex determination of European sea bass (34, 69, and 132 dph) (Fig. 1). Fish were also measured and weighed at 73 dph to monitor early growth rate. We analyzed glucocorticoids in whole fish as well as the expression of key genes coding for the previously mentioned enzymes involved in the stress response in fish (*gr1*, *gr2*, *mr*, *cyp11b*, *hsd11b2*, *mc2r*, *crf*, *pomc*, *star*, and *cyp21a2*) and the level of monoamines in the telencephalon of juveniles undergoing molecular sex differentiation^31,32^. Finally, by using a predefined breeding plan, we assessed the influence of breeder origin (wild or selected for growth), on the effects of the experimental treatments on fish sex ratio.

## Results

### Cortisol in the water

The slight increase of temperature (0.5 °C/day) triggered cortisol production in the two conditions (Fig. 1), as expected. We also detected significant (*p* < 0.001) increased cortisol production over time (*i.e.* larval development). During the whole cortisol monitoring period there was on average 3 times less cortisol in the water (ng/L) in the low-density treatment when compared to the control group (*p* < 0.01) (Fig. 1A), confirming the accuracy of our test, since there was 3.3 times less individuals in this condition. When focusing only on the period of temperature increase, cortisol production per fish (pg/L/fish) was about 2.5 times lower in the low-density treatment when compared to the control group (*p* < 0.05) (Fig. 1B).

### Whole-body glucocorticoids and progestogens

To depict the importance of cortisol when compared to other hormones, we performed comparisons independently of the treatment. Overall, there was about 3.4 times more cortisol than 17α-hydroxy-progesterone (Figure S1A), 2 times more cortisol than 11-Deoxycortisol (Figure S1B), 2.2 times more cortisol than cortisone (Figure S1C) and 6 times less cortisol than tetrahydrocortisone (Figure S1D), although this highly depended on the stage, with a decrease of the ratio as fish got older, except for cortisone that remained relatively stable (Figure S1).

Since individuals in the tryptophan condition did not start to feed on the supplemented diet before 40 dph, and because no differences were observed between cortisol release in the water by fish from this and the control group, we pooled samples from both experimental groups at 34 dph for statistical analysis. There was no difference between treatments for any of the hormones evaluated.

### Trunk and head gene expression

**Figure 2:**
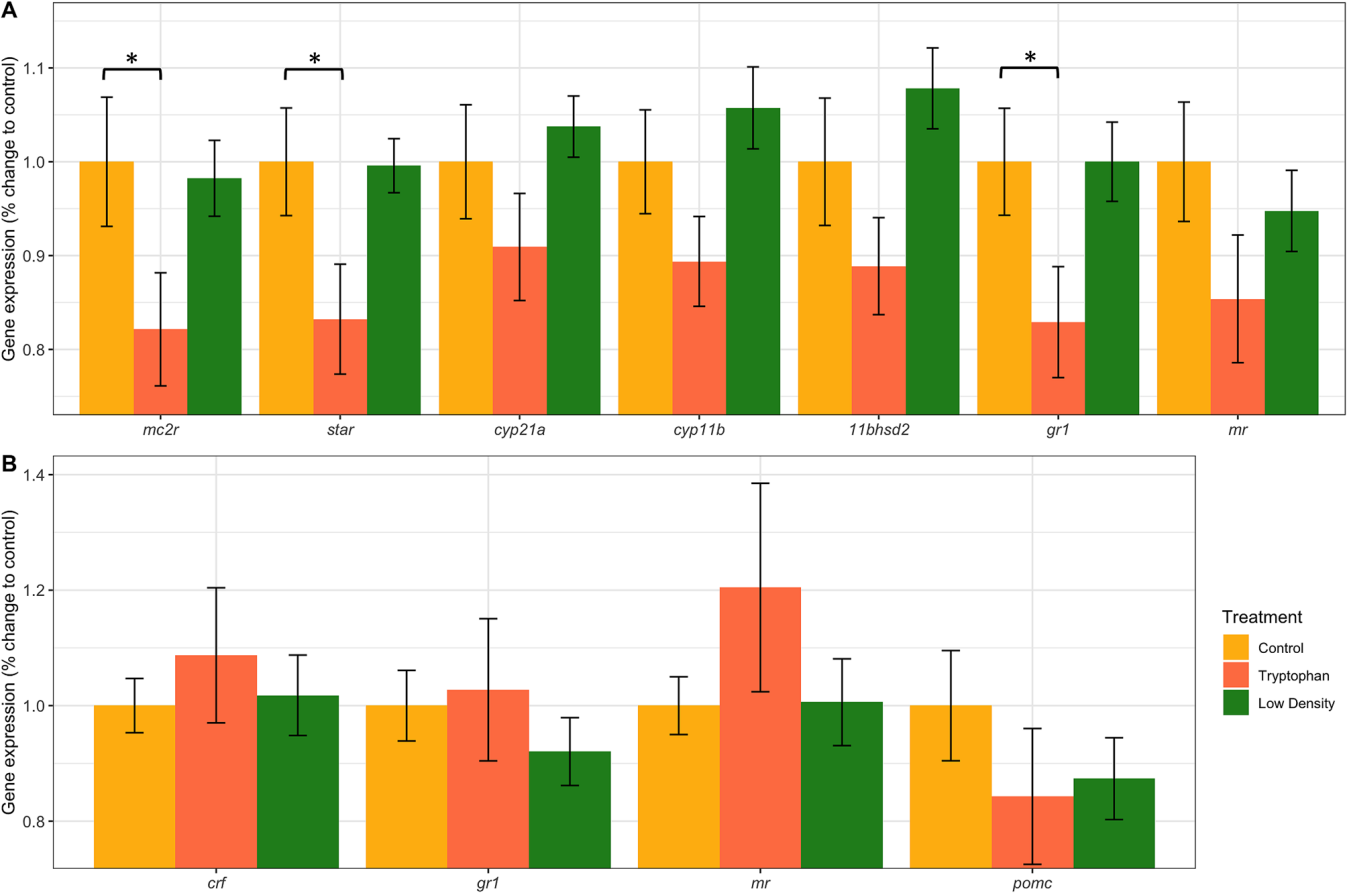
Gene expression of (**A**) *mc2r, star, cyp21a, cyp11b, hsd11b2, gr1 and mr* in the whole trunk of juveniles and (**B**) of *crf, gr1, mr* and *pomc* in the head of juveniles kept in the 3 different treatments: Control, Low density and Tryptophan. Note that these are values relative to the geometric mean of the following reference genes: ribosomal protein L13 (*L13*) and eukaryotic translation elongation factor 1 alpha (*eef1a*). Each bar plot represents the difference in % between Control and the other treatments. “*” indicates a *p* value < 0.05.

At 69 dph, fish fed a tryptophan-supplemented diet showed significantly lower expression levels of *mc2r, star*, and *gr1* in the trunk than those from the control group (Fig. 2A). The *crf, gr1, mr*, and *pomc* expression levels measured in the head were similar between the different experimental treatments (Fig. 2B).

### Fish size and number at the all fins and juvenile stage

At 73 dph, fish (a subsample of 30 individuals from each tank) from the low-density treatment were significantly (*p* < 0.05) heavier (0.33 ± 0.1 g) and longer (3.06 ± 0.4 cm) than those from both the control (0.29 ± 0.1 g; 2.94 ± 0.4 cm) and tryptophan (0.28 ± 0.1 g; 2.83 ± 0.4 cm) treatments. Individuals from the tryptophan treatment were significantly shorter than the control group (*p* < 0.05). Total fish number at 73 dph was estimated to be 1370, 1870, and 2020 individuals in the low-density, tryptophan, and control treatments, respectively. At that point we re-adjusted the density to 1.8 fish/L (200 fish/tank) in the low-density treatment and to 7.3 fish/L (800 fish/tank) in both the control and the tryptophan treatments. At 132 dph, the weight and length of sampled fish (n = 20 per treatment) were similar between all treatments.

### Monoamines in telencephalon and gene expression in hypothalamus

**Figure 3:**
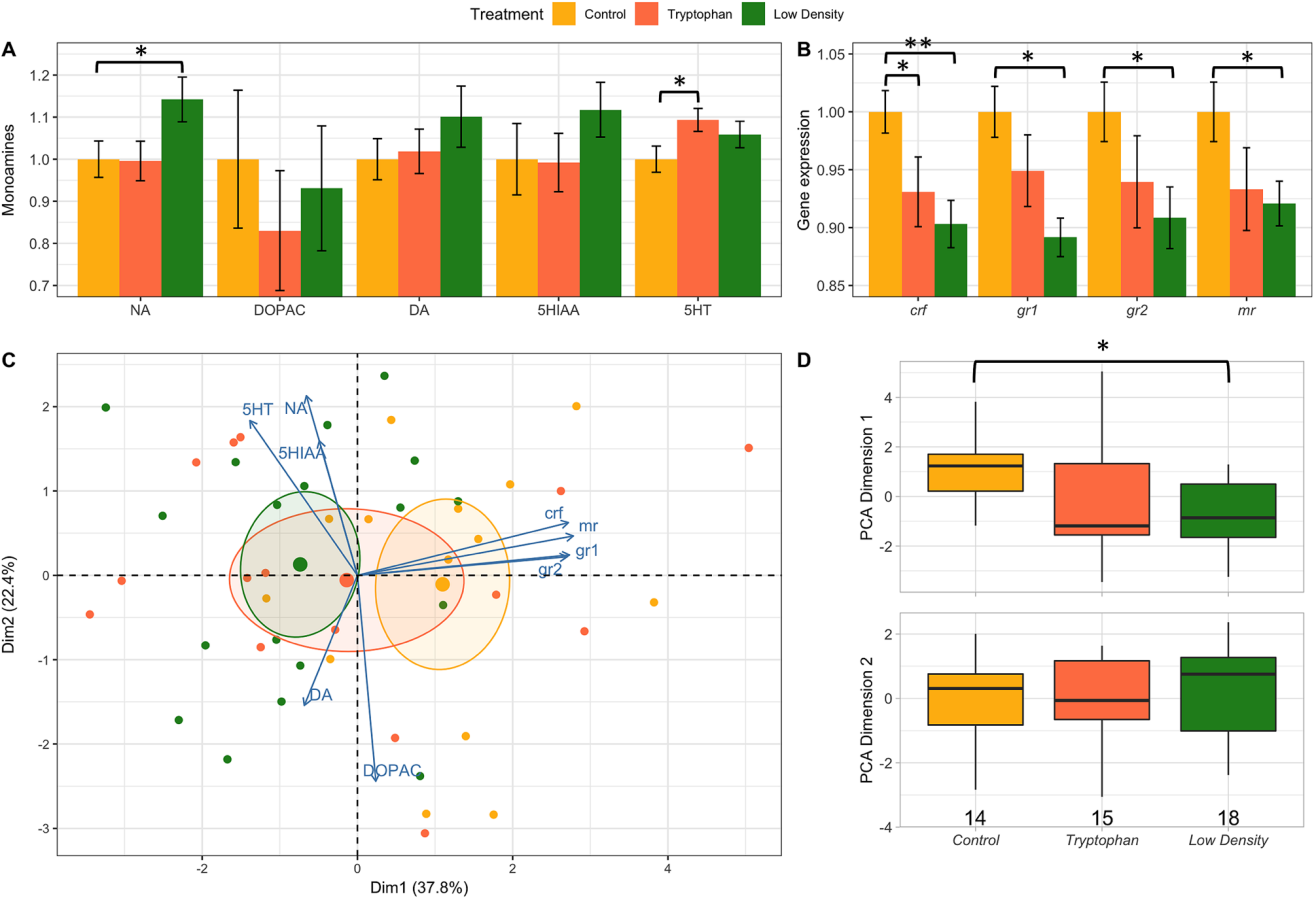
Overall view of (**A**) monoamines detected in the telencephalon and (**B**) stress-related genes detected in the hypothalamus (*crf, mr, gr1, gr2*) of fish sampled at 132 dph in the 3 treatments: Control, Low density and Tryptophan. Note that gene values are relative to the geometric mean of the following referencegenes: ribosomal protein L13 (*L13*) and eukaryotic translation elongation factor 1 alpha (*eef1a*). Each bar plot represents the difference in % between Control and the other treatments. (**C**) Represent output of the Principal Component Analysis (PCA), based on monoamines and gene expression in each individual. (**D**) The coordinates of each individual within each treatment for the first and the second axis are represented by box plots that show the median, which is represented by lines, and the first and third quartiles. “*” indicates a *p* value < 0.05 and “**” indicates a p-value < 0.01.

At 132 dph, significantly more norepinephrine (NA) was detected in the telencephalon of the fish from the low-density treatment compared to those of the control group (Fig. 3A). In addition, more serotonin (5HT) was observed in the telencephalon of fish from the tryptophan treatment compared to the control group (Fig. 3A).

At 132 dph, there were significantly (*p* < 0.05) less transcripts of *crf, gr1, gr2,* and *mr* in the hypothalamus of fish kept at low density when compared to the control group (Fig. 3B). There were also significantly (*p* < 0.05) less transcripts of *crf* in the tryptophan condition compared to the control group (Fig. 3B). We ran a PCA with all monoamines and genes (since they were all measured on the same fish) (Fig. 3C). The first axis (38% of variance explained) allowed clearly distinguishing fish that presented high level of expression of stress related genes (Fig. 3C). The second axis (22% of variance explained) was more related to monoamine concentration. By extracting the coordinates of the Dimension 1 and 2 of the PCA, we detected that fish from the low-density treatment significantly differed from the control group in the dimension 1 (*p* < 0.05) (Fig. 3D). Overall, dimension 1 allowed distinguishing between the low density and the control treatments (Fig. 3D).

### Parentage recovery

From the 96 SNP submitted, 86 were successfully genotyped by the KASP technology. DNA extraction was unsuccessful for 3 sires (2 from the wild group and 1 from the selected line) and 2 additional wild sires displayed a poor-quality genotyping (with a call rate < 20%). A total of 1027 fish were assigned to their specific parental pair. Among the successfully assigned fish, only 193 were assigned to a wild sire while 834 were assigned to a selected sire.

### Sex-ratio and weight difference

**Figure 4:**
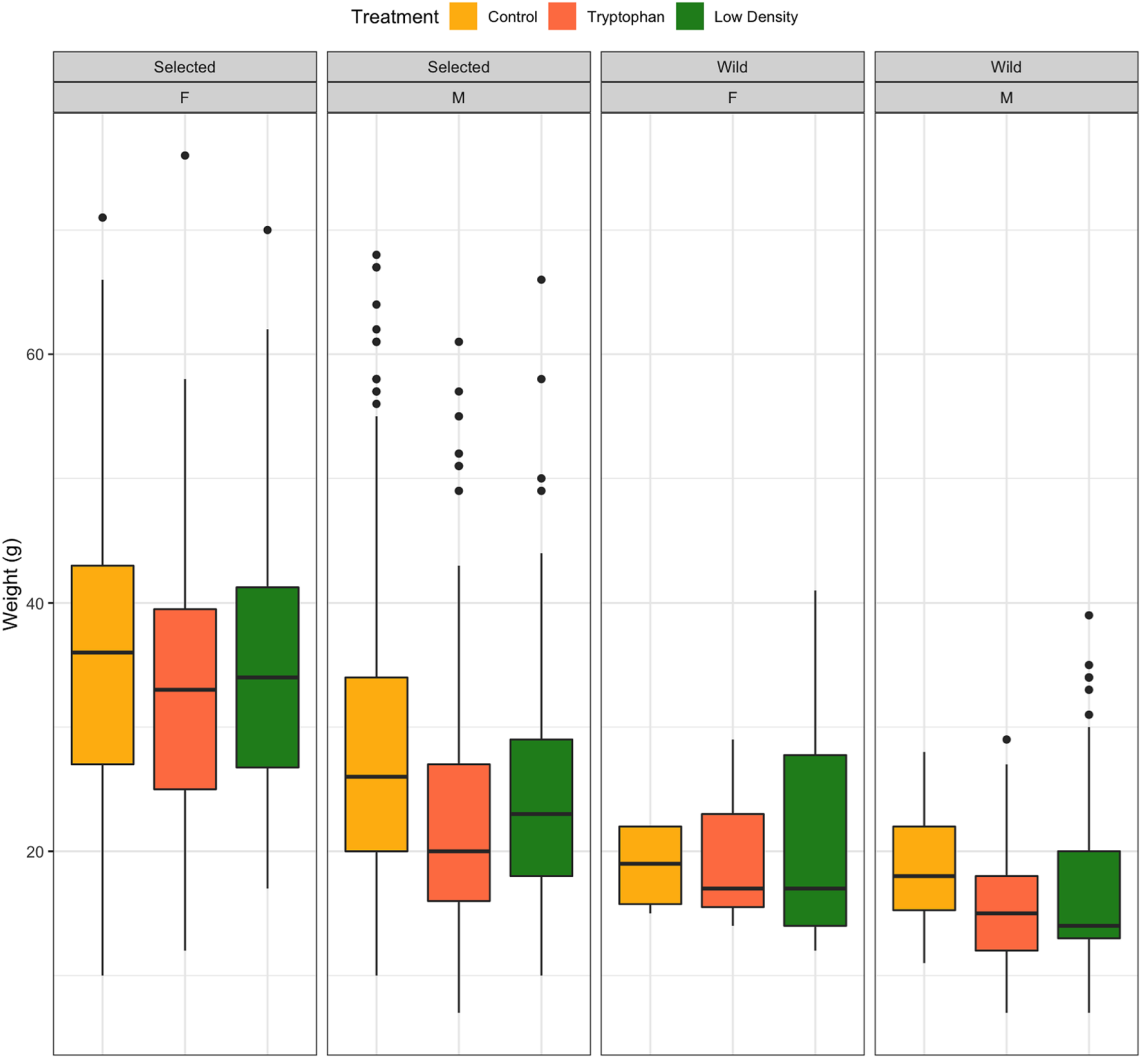
Weight (g) at 215 dph of individuals from the 3 treatments: Control, Low density and Tryptophan according to their sex (male, M or females, F) and genetic origin (wild *vs* selected). Box plots show the median represented by lines, and the first and third quartiles as well as outliers are represented by full circles outside the box.

**Figure 5:**
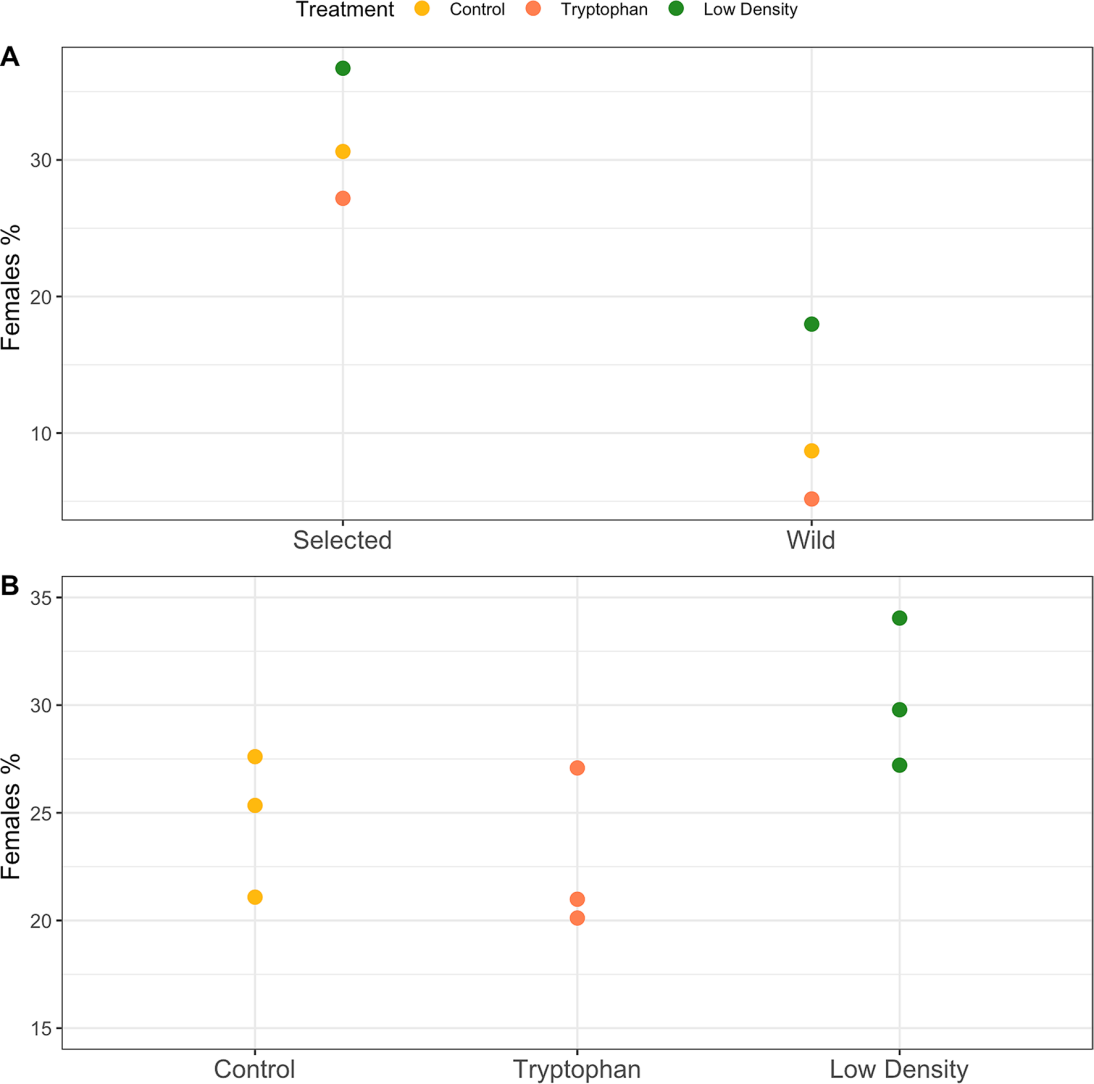
Sex-ratio at 328 dph of fish from the 3 treatments: Control, Low density and Tryptophan according to (**A**) genetic origin (wild *vs* selected) and (**B**) treatments with the tank replicates (n = 3) represented.

At the end of the treatment period (215 dph), fish weight significantly differed between sires’ origin (wild or selected), sex (female or male; assessed at 328 dph) and treatment (Fig. 4). None of the interactions between terms were significant. The fish from selected parents displayed higher growth than those from wild parents (*p* value < 0.001; Fig. 4). Females displayed higher growth than males independently of their genetic origin (p-value < 0.001; Fig. 4). The weight of the fish from the control group at 215 dph was higher than that of the other treatments (*p* value < 0.001; Fig. 4). On average, and independently of the treatment, more females were detected in fish whose parents had been selected for growth (30%) when compared to fish from wild parents (10%, *p* < 0.001, Fig. 5A). Interestingly, the low-density treatment was the one providing more females in both cases. Overall, the sex-ratio differed between treatments (F-value = 5.0189, *p* < 0.001) with more females (*p* < 0.05) in the low-density when compared to the control group (Fig. 5B), while treatment × origin interaction was not significant.

## Discussion

Overall, parents from the strain selected for growth produced more females than those of wild origin. Fish kept at low density exhibited a high early growth rate and produced less cortisol following a temperature increase when compared to the control larvae. In addition, expression of key stress-related genes in the hypothalamus was downregulated compared to the control group during molecular sex determination. At the end of the experiment, more females were produced when individuals were kept at low density, independently of the genetic background, pointing to a potential mechanistic link between stress and/or growth and a higher female differentiation rate.

The monitoring of cortisol release in the water at the earliest stages of development showed that this method was useful for detecting (1) differences between treatments and (2) the effect of a stressor (temperature). As expected, there was less cortisol in the water of fish kept at low density when compared to the control group (ng/L), but interestingly, it was also the case when controlling for fish number (pg/L/fish) during the specific increase of temperature, highlighting that individuals of the low-density treatment also produced less cortisol at this stage. One might wonder if temperature really triggers an increase of cortisol production or whether this is due to a developmental process in which bigger individuals produce more cortisol, as previously demonstrated in sea bass^33,34^. However, these two studies did not provide values at such a short time scale (i.e. day) and, in addition, they showed discrepancies in their results; one highlighted an increase of the whole-body cortisol between the first feeding stage (11 dph) and the flexion stage (27 dph)^33^, while the other observed a decrease ^34^. Here, using water cortisol, we clearly show that the rise of cortisol occurs very readily in the two days following the increase of temperature, and such a quick increase could not be merely attributed to fish growth in this brief period of time. Temperature has been shown to increase cortisol production in several fish species^35^ and, by modeling the metabolic increases of cortisol linked to temperature, we previously showed that this small temperature rise (0.5 °C/day) likely triggered a real physiological stress, independently of the fish size, and could not be merely attributed to metabolic adjustments to warmer tempertures^36^.

At later stages, but with the same temperature (i.e. 19 °C), we also noted an increase of whole-body glucocorticoid content with age, with for instance, cortisol concentration (ng/g) being 85 times higher at 69 dph when compared to 34 dph. However, we did not detect any differences between the low-density and the control treatment for any of the hormones measured. One possible explanation for this lack of consistency in the effects of low stocking density might be related to the sample numbers and techniques for measuring cortisol. When measuring cortisol in the water, we averaged the release of all the fish in the tank, which resulted in a relatively low variation than when compared to fish and small pools. Still, other studies showed a relatively good correlation between water-borne and whole-body cortisol^37–40^, so that the most parsimonious explanation is likely that the differences observed at the earliest stages do not exist anymore as fish grow, probably because differences in fish density between the control and the low-density treatment were not as high at 69 dph (18 vs. 12 fish/L at 73 dph) when compared to the earliest larval stages (estimated at 125 vs. 30 fish/L after hatching).

Once the density was re-adjusted at 73 dph, we detected significant changes between the control and low-density treatments in the expression of all genes involved in stress regulation in the hypothalamus of juveniles. The down-regulation of *crf*, *gr1*, *gr2,* and *mr* is in agreement with previous findings in medaka (*Oryzias latipes*) and bluehead wrasse (*Thalassoma bifasciatum*), that showed a down-regulation of both *crf*^41^ and *gr1*^15^, where these genes were shown to play a critical role in sex reversal. Interestingly, this occurred at the exact time (132 dph) when the gonad is expected to be in the process of sex differentiation^31,32^, suggesting a link between stress regulation and sex determination, though further investigation is needed at the individual level. One might be tempted to take these reduced mRNA expression as corresponding to less-stressed fish; however, these results should be interpreted with caution. Both GRs and MR are involved in physiological and behavioral stress responses^42,43^, but their regulation is complex and their mRNA expression and responsiveness to stress varies depending on the brain area/peripheral tissue and the species considered^44,45^. For instance, exposure to a chronic stressor triggered a down-regulation of these receptors in the brain of the common carp (*Cyprinus carpio*)^46^ and in the preoptic area of the Atlantic salmon (*Salmo salar*)^47^. However, this long term exposure to a stressor also triggered an up-regulation of those same glucocorticoid receptors in the pituitary gland of the Atlantic salmon^47^ and of *crf* in the hypothalamus of the common carp^48^. Finally, rearing densities did not affect gene expression of glucocorticoid receptors in the telencephalon of rainbow trout (*Oncorhynchus mykiss*)^49^.

The observation of more females in the low-density treatment when compared to the control treatment contradicts a previous study on European sea bass that failed to find sex-ratio differences between high and low-density treatments (from 10 to 26 times less fish at low density) for fish kept between 13 and 126 dph under the two conditions^27^. In the experiment of Saillant et al.^27^, they started with 200 larvae/L vs. 20 larvae/L and observed on average 12% of females in both conditions, while in the present study, the difference was even lower after hatching (125 larvae/L vs. 30 larvae/L) and at 73 dph (18 fish/L vs. 12 fish/L). Excepting the initial fish density, one major difference relies on the fact that our fish were almost not disturbed throughout the whole process to minimize stress (only one size-grading at 73 dph to adjust density), while in that previous experiment^27^ fish were regularly size-graded. This may even explain why our control condition produced more females (25%) than the control group of Saillant et al.^27^. Our results, however, are concordant with those found in the sea lamprey (*Petromyzon marinus*)^50^, zebrafish^7^, temperate eel species^6^, and pejerrey (*Odontesthes bonariensis*)^51^ with more males detected at higher densities. Interestingly, in fish from both selected and wild parents, the low-density treatment was the one producing more females, showing that this effect is not influenced by the genetic background, and that sea bass would likely be a GSD + ESD (not TSD) species.

As expected, more females were produced by parents selected for growth when compared to wild parents in our experiment. There is a positive genetic correlation (+ 0.50) between the tendency to produce females and body weight^21^ and thus, selection for growth is expected to generate a correlated response on sex-ratio towards more females^52^. Note that in the present experiment, the genetic difference between groups was generated by crossing wild or captive males with wild females, so that the observed difference between groups was only half of the expected additive genetic effect between the selected and the wild population. A question remains on whether the effect of growth on sex ratio is linked to the phenotypic consequences of fast growth, with fast growers differentiating as females, irrespective of the cause (genetic or environmental) of fast growth.

Fish kept at low density exhibited a faster growth rate between 0 and 73 dph when compared to the control group, and this difference was then not noticeable at 132 dph (even though the differences in density were stronger), which is concordant with previous studies on sex-related growth^28,53^. We could thus not discard the fact that early growth rate, instead of density per se or stress*,* would be key in determining the sex of individuals, as this hypothesis has been already proposed for sea bass^28,53,54^ and other species, where density interacts with growth^6^. Stress and growth have often been linked, with stressed individuals having lower capacity to invest energy for somatic growth^55^, so that the alternative hypothesis, meaning that slower growth rate results in masculinization, might also hold true. In this sense, sex-reversal of genetic females of medaka (*Oryzias latipes*) undergoing a period of fasting was recently highlighted^56^ and the hypothesis of energy limitation deserves to be further investigated in sea bass.

Results obtained from individuals fed the tryptophan-supplemented diet possibly evidenced another pathway in which the serotonin system would alleviate the potential effects of diminished stress on sex determination. This group was kept at the same rearing density as the control group and, since dietary tryptophan supplementation has been shown to function as a stress-mitigating strategy in different fish species^19,57^, we expected this treatment to reduce the stress status of fish compared to the control group. Stress-mitigating effects of tryptophan seem to be mediated by increased production of brain serotonin and/or pineal melatonin, tryptophan being a precursor for both compounds. In the current study, tryptophan supplementation seemed to be effective, since serotonin level was increased in the telencephalon at 132 dph. Additionally, this group displayed a lower growth rate than the control group during the first month of feeding with the tryptophan-supplemented diet. Tryptophan and its derivatives, serotonin and melatonin, have shown to have inhibitory effects on fish growth^58–60^. Indeed, serotonin influences both brainstem reflex centers and hypothalamic integratory centers involved in controlling food intake. There is an inverse relationship between the level of brain serotonin signaling and food intake, that is when brain serotonin signaling is augmented, food intake is reduced, and vice versa^61^. The coregulation of serotonin and *crf* gene expression detected in fish from the tryptophan treatment evidenced a close relationship between the HPI axis and the brain serotonin system in sea bass. Since serotonin and stress-related gene expression levels, including *crf*, indicated a reduced stress status in fish fed the tryptophan-supplemented diet, a higher female:male ratio than in the control group was also expected. However, both tryptophan and control groups presented the same sex ratio. We suggest that the activation of the serotonin system in the tryptophan group during the sex differentiation window may have attenuated the expected female-promoting differentiation effect of the reduced stress level, as serotonin was shown to promote masculinization in other fish species. For instance, the treatment of Mozambique tilapia (*Oreochromis mossambicus*) with a serotonin synthesis inhibitor resulted in a significant increase in the proportion of males^62^. Moreover, in African sharptooth catfish (*Clarias gariepinus*), differential expression of tryptophan hydroxylase 2 (tph2), the rate-limiting enzyme in the synthesis of neuronal serotonin, was observed at mRNA and protein levels in the brains during development, with higher levels in males than in females, which further correlated with the 5-hydroxytryptophan and serotonin brain levels^63^. A dimorphic expression of *tph* during early brain development around the critical period of gonadal sex determination was also observed in the Nile tilapia (*O. niloticus*), also indicating a role of serotonin in brain sex differentiation in this species^64^. The same authors observed that *tph* was male brain-specific (in the telencephalon-preoptic area) during early development (at 12 dph) and the changes in *tph* expression agreed with the levels of serotonin in the preoptic area-hypothalamus^65^. Reciprocal interactions between central CRF and serotonergic systems are involved in modulating reproductive and social behaviors in many vertebrates, including fish^66,67^; and our findings hint at a possible communication between these two systems also during sex determination. However, since serotonin also inhibits feed intake, the question about whether sex determination is first driven by hormonal signaling and/or by their effects on growth rate at early developmental stages remains to be solved.

Although our results provide only correlations between stress (cortisol and serotonin) and sex at the group level, they provide for the first-time evidence that other environmental factors than temperature can affect the sex of European sea bass. Future studies should thus focus on collecting data at the individual level to clearly detect the possible link between cortisol, serotonin, feed intake, growth, and sex determination in this species.

## Methods

We designed a specific system with 3 recirculating aquaculture systems (RAS), each with 4 tanks connected to a common biofilter tank, so that each condition (tryptophan, low-density and control) had 4 replicates.

### Experimental fish

Larvae were produced by artificial fertilization^68^. To assess the impact of selection for growth on sex ratio, ovules from 16 wild Western Mediterranean dams were fertilized with cryopreserved sperm from two types of sires in a separated full factorial design: 20 wild Western Mediterranean sires and 19 captive sires from Western Mediterranean origin that are the result of three successive generations of individual selection for growth. All fish were reared at the experimental aquaculture station of Ifremer (Palavas-les-Flots, France). Seventy-two hours after hormonal induction of ovulation (10 µg/kg luteinizing releasing hormone, Sigma D-TRP6LHRH), females were manually stripped and 100 ml of eggs from each of the 16 females were collected, and mixed to create a 1600 ml pool of eggs, in which we sampled 39 aliquots of 20 ml each. Each aliquot was then fertilized by thawed cryopreserved sperm from a single sire. In total, 20 wild sires and 19 selected sires were used. One minute after activation of the sperm with sea water, fertilization batches were pooled by sire origin, and each origin was split in two replicate incubation tanks at 14 °C.

### Experimental design

**Figure 6:**
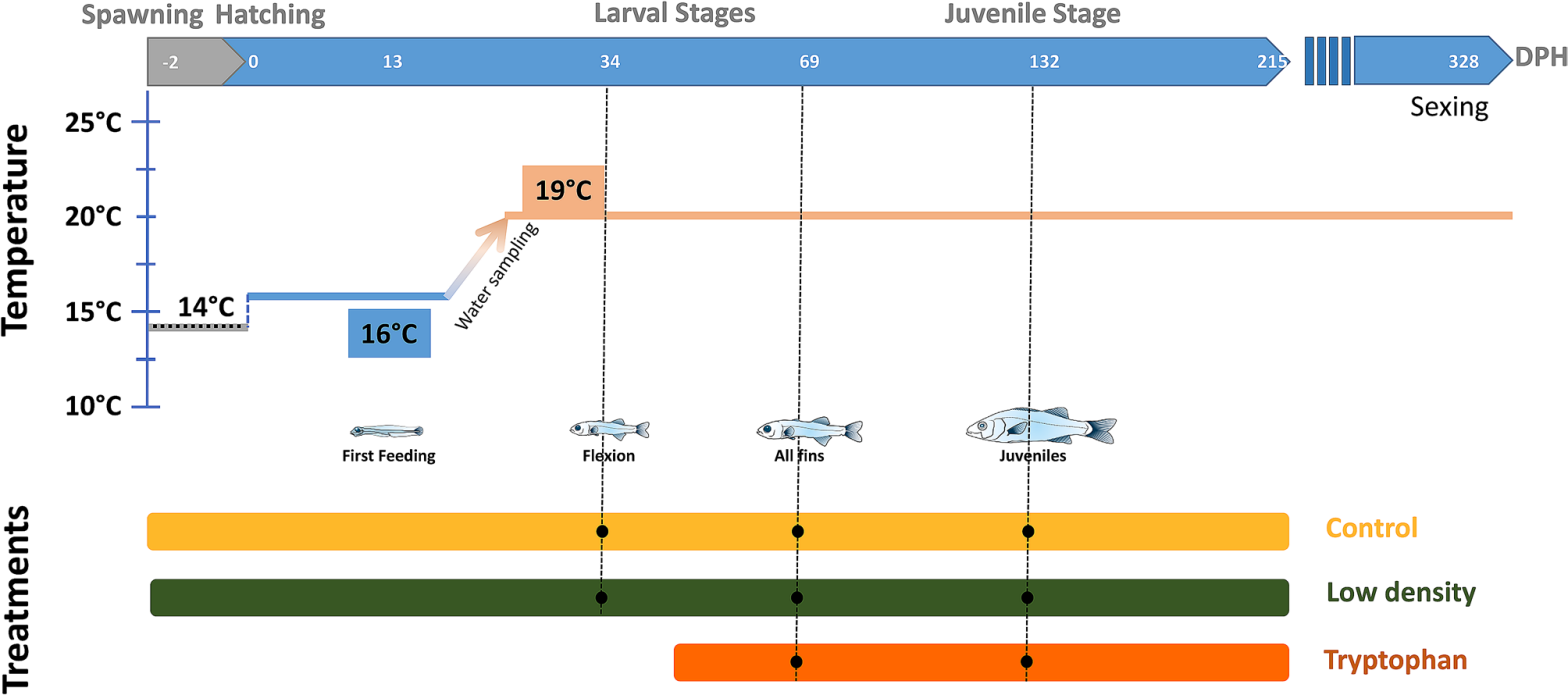
Schematic representation of the experimental design for fish sampled at 3 different time (34, 69 and 132 dph) and water during the increase of temperature for the 3 different treatments: Control, Low density and Tryptophan.

At 48 h post-fertilization, floating (live) eggs were dispatched in 12 110-L tanks (50 cm depth) at stocking densities of 150 eggs/L for the control and the tryptophan conditions, and 38 eggs/L for the low-density condition. Hatching rate was estimated on 72 eggs from both selected and wild origin and kept in seawater in 24-well plates. Hatching rate was 81% for fish from selected origin and 82% for fish of wild parents. We thus estimated the initial density to be 125 larvae/L in the first two conditions and 30 larvae/L for the low-density condition. Between hatching and 2 dph, temperature was gradually increased from 14 °C to 16 °C and larvae were kept in the dark for the first 10 days (Fig. 6). From 10 dph onwards, the light (AquaRay miniLED 500, 10000K white, Tropical Marine Center) was turned on (12L/12D). Larvae were fed *Artemia* nauplii from 10 to 40 dph.

The temperature-increase protocol started at 17 dph and 16 °C, with a progressive increase of 0.5 °C per day until reaching 19 °C at 22 dph. Temperature was monitored in the biofilter tank twice a day throughout the experiment to avoid disturbing the fish. We minimized all possible sources of perturbation (e.g. no swim-bladder sorting was performed, daily husbandry tasks were carried out by a single person, ensuring minimum noise) and larvae were fed *Artemia* using an automated peristaltic pump delivering food continuously. At 40 dph all fish were weaned onto a commercial sea bass diet (Pro Start and Pro Wean, BioMar, Nersac, France) with automatic feeders ensuring that they were fed ad libitum. From that point onwards, the tryptophan treatment started. The same commercial diet was supplemented with 3% (dry mass) of tryptophan at INRAE St Pée sur Nivelle, France. The supplementation was performed with 1.37% refined rapeseed oil. At 73 dph, actual fish density and mortality were calculated. All control and tryptophan tanks were adjusted to 800 fish and the low-density treatment was adjusted to 200 fish per tank. We also measured and weighed (mean ± sd) a subsample of 30 fish per tank that were then euthanized. The mortality rate between hatching and 73 dph was 86% for both the tryptophan and the control treatment, and 60% for the low-density treatment.

### Water sampling for cortisol measurement

Total water volume of each RAS was ~ 700 L, and water was renewed at a flow of 100 L/h. All experimental tanks included an air stone to ensure oxygen supply and removal of carbon dioxide from the water. At 1 pm, the open water system (delivering new seawater) was closed for 1 h, so that each RAS was fully closed (as in Geffroy et al.^69^). For every sampling, at 2 pm, three 500-mL samples of water were collected in new plastic beakers from the 4 tanks sharing the biofilter (n = 12 × 500 mL at each time). The 500-mL beakers were then stored at -20 °C before being processed all at once. Water was sampled with a similar protocol at 7 different time-points: 13, 17, 18, 19, 20, 21, and 28 dph. Cortisol extraction was performed using C18 solid phase extraction cartridge (Sep-pak Plus C18, Waters Ltd.; www.waters.com), as previously described^69,70^. The data of control and tryptophan conditions were pooled, since larvae were not yet fed the tryptophan enriched diet.

### Larval sampling for cortisol measurement

At the ‘flexion’ stage (34 dph) and the ‘all fins’ stage (69 dph), a sub-sample of larvae was quickly collected (from only one tank per treatment to avoid stress in the 3 other tanks used for sex-ratio assessment) and flash-frozen to avoid any release of cortisol (Fig. 6). For each RAS, 10 pools of 3 individuals were used to quantify hormones at 34 dph and 8 individuals (processed individually) were sampled at 69 dph. Each pool or individual was rinsed with distilled water, dried on paper tissue, transferred into a 1.5-mL Eppendorf tube using tweezers, and stored at -20 °C. Larval tissue was then homogenized and 2 mg of sample was transferred into a 12-mL tube. Cortisol was extracted from each sample using 8000 µL of methanol (HPLC grade) and 1 µL of a cortisol-d4 solution of 0.5 µg/L as internal standard. Each sample was vortex-mixed for 30 s until homogenization and placed on an overhead shaker for 60 min at 90 rpm. Subsequently, each sample was centrifuged for 10 min at 3452 g (= 4000 rpm on a swing-out) at 7 °C and the supernatant was transferred to a new 12-mL tube. Each sample was evaporated to dryness under nitrogen at 60 °C and resuspended in 5000 µL H_2_O/MeOH (80:20; v/v). After conditioning a C18 SPE column (C18-Max, 500 mg, 6 mL) with 3 mL of MeOH (HiPerSolv) followed by 3 mL of H_2_O (Type I), each sample was loaded. The column was washed with 4.5 mL H_2_O/MeOH (65:35; v/v) and retained compounds eluted with 2.5 mL H_2_O/MeOH (20:80; v/v) into a 12-mL test tube and evaporated to dryness under a stream of nitrogen at 60 °C using a nitrogen evaporator.

### Hormone quantification

Cortisol in the water was analyzed by competitive ELISA following manufacturer’s recommendations (Neogen Lexington, HI, USA) and as previously described^36^. For whole larvae, each processed sample was reconstituted in 50 µL H_2_O/MeOH (80:20; v/v) in a vial with insert and analyzed by UPLC-MS/MS (Xevo TQS, Waters, Milford, USA) using an Acquity UPLC BEH C18 (1.7 µm; 2.1 mm × 100 mm) chromatographic column. Glucocorticoids were assessed in these samples by comparison with an internal standard; however, the obtained internal standard area was not representative for fishes at 69 dph and we thus did not use absolute quantitative levels. Instead, relative differences between all conditions and controls (%) were calculated, as all samples were analyzed in the same batch.

### Gene expression

At the ‘all fins’ stage (69 dph), a sub-sample of 10 individuals per treatment was sampled (in only one tank) for analysing the expression of genes involved in the stress cascade. Fish were euthanized using benzocaine (150 mg/L), the head was then quickly separated from the trunk and placed in individual 1.5 mL RNAse free tubes, which were flash-frozen in liquid nitrogen and placed at − 80 °C until further processing. At 132 dph, 20 fish per condition were also sampled. They were quickly euthanized using benzocaine (150 mg/L), and the brain of each individual was removed to extract the hypothalamus (gene expression) and the telencephalon (monoamines, see next section). Those parts of the brain were processed similarly in 1.5 mL tubes and placed at − 80 °C until further processing. The trunk, the head and the hypothalamus were individually grinded using NucleoMag RNA kit following manufacturer’s instructions (Macherey–Nagel). We used a KingFisher Flex automatic extraction robot with all reagents provided in the NucleoMag RNA kit (Macherey–Nagel) for the RNA extraction of all samples.

RNA quantity was assessed using a NanoDrop ND-1000 V3300 Spectrophotometer (Nanodrop Technology Inc., Wilmington, DE, USA). Complementary DNAs (cDNA) were synthesized using the GoScrip Reverse Transcription System kit following manufacturer’s instructions (Promega, Madison, WI, USA) with a starting quantity of 1 μg of total RNA as previously described^71^. All cDNAs were diluted 10 folds in nuclease-free water prior to quantitative real-time PCR (qPCR) analyses.

The *mr, gr1, gr2, cyp11b, mc2r,* and *crf* primers were taken from the literature^34,71–73^, while *hsd11b2, pomc, star,* and *cyp21a2* primers were designed using Geneious Prime software from the published (annotated) genome of the European sea bass^74^ (Table 1). The cDNA product of these genes was then sequenced, confirming their identity. We used the following reference genes: ribosomal protein L13 (*L13*) and the Eukaryotic translation elongation factor 1 alpha (*eef1-alpha*)^71^. Primer sequences and efficiencies are provided in Table 1. An Echo 525 liquid handling system (Labcyte Inc., San Jose, CA, USA) was used to dispense 0.75 μL of SensiFAST SYBR No-ROX Kit (Bioline, London, UK), 0.037 μL of each primer, 0.21 μL of ultra-pure water and 0.5 μL of diluted cDNA into a 384-well reaction plate. Each sample was run in duplicate. The qPCR conditions were as follows: denaturation at 95 °C for 2 min, followed by 45 cycles of amplification (95 °C, 15 s), hybridization (60 °C, 5 s) and elongation (72 °C, 10 s), and a final step at 40 °C for 30 s. Relative levels of gene transcription were obtained using the following equation (2^(Ct_ref))/2^(Ct_target) with the target gene normalized by the geometric mean of two genes as reference (*L13* and *eef1-alpha*).

**Table 1:** Primer sequence, amplicon size and efficiency of all genes used in the study.

Gene	GenBank accession number	Primers	Primer sequence 5' to 3'	Amplicon size (bp)	Efficiency	Reference
*gr1*	AY549305.1	gr1-F	GAGATTTGGCAAGACCTTGACC	401	2	Pavlidis et al.^33^
		gr1-R	ACCACACCAGGCGTACTGA			
*gr2*	AY619996	gr2-F	GACGCAGACCTCCACTACATTC	403	2.2	Pavlidis et al.^33^
		gr2-R	GCCGTTCATACTCTCAACCAC			
*mr*	JF824641.1	mr-F	GTTCCACAAAGAGCCCCAAG	197	2.12	Sadoul et al.^71^
		mr-R	AGGAGGACTGGTGGTTGATG			
*cyp11b*	AF449173.2	cyp11b-F	GGAGGAGGATTGCTGAGAACG	80	2	Socorro et al. 2007
		cyp11b-R	AGAGGACGACACGCTGAGA			
*hsd11b2*	DLAgn_00250230	hsd11b-F	CAGGCACGTTACTTCGCTGG	141	2	Goikoetxea et al. ^36^
		hsd11b-R	TGACTGCTTCCTTAGAGCGC			
*mc2r*	AJ866727.1	mc2r-F	CATCTACGCCTTCCGCATTG	80	1.95	Tsalafouta et al. 2017
		mc2r-R	ATGAGCACCGCCTCCATT			
*crf*	DLAgn_00076040	crf-F	TGACCTCACAGACTACCT	217	1.95	Present study
		crf-R	GTCAGGTCCAGGGATATCGG			
*pomc*	DLAgn_00069720	pomc-F	GGATACTGGACTGTATTCACCT	291	1.82	Present study
		pomc-R	GAAATGCCCTCAGAAGATCC			
*star*	DLAgn_00115570	star-F	TGAGCTGAACAGACTGGCAG	216	1.94	Present study
		star-R	TCTCCATTCGCAGCCACAAT			
*cyp21a2*	DLAgn_00061920	cyp21a-F	ACAGCACTGCCTTGATAGTCC	123	1.92	Present study
		cyp21a-R	GGCACATCGTCGTCTTGTTC			
*L13*	DLAgn_00023060	L13-F	TCTGGAGGACTGTCAGGGGCATGC	148	2.1	Sadoul et al.^71^
		L13-R	AGACGCACAATCTTGAGAGCAG			
*ef1-*α	AJ866727.1	ef1-F	AGATGGGCTTGTTCAAGGGA	166	1.97	Sadoul et al.^71^
		ef1-R	ACAGTTCCAATACCGCCGA			

### Monoamines

The telencephalon of each fish was sent in dry ice to the Technical University of Denmark where they were processed for HPLC analysis of monoamines. Tissues were weighed and immediately homogenized in 1 mL of a Perchloric acid solution (0.4 M Perchloric acid and 0.1 mM EDTA) with a Sonopuls ultrasonic homogenizer (Bandelin, Germany). Homogenates were then centrifuged for 10 min at 14,000×*g* (at 4 °C). Supernatants were diluted 1/20 in the PCA solution and 20 µL of the dilution was analysed using high performance liquid chromatography coupled to electrochemical detection (HPLC-EC) as previously described^75^. The levels of serotonin (5HT), dopamine (DA) and their main oxidative metabolites 5-hydroxyindoleacetic acid (5HIAA) 3,4-dihydroxyphenylacetic acid (DOPAC) were quantified, along with the levels of norepinephrine (NA).

### Growth rate and sex assessment

At the end of the experimental period (215 dph), fish were anaesthetised (45 mg/L benzocaine); the remaining fish of the low-density treatment (n = 546) and 190 fish per tank of the control and the tryptophan treatment were randomly sampled, for a total of 1686 fish. Those fish were individually measured, weighed, and tagged with RFID glass tags (Biolog-ID, France), keeping their initial tank identification. They were then moved to four tanks of 1500 L, mixing groups (420 fish in each 1500 L tank). In each tank, 180 randomly chosen fish of the same size were added to obtain a final density of 0.4 fish/L. This redistribution was done to save space, and was not supposed to impact the sex-ratio as fish were histologically differentiated at that time (mean size = 11.5 cm)^23^. Following individual tagging, several fish died in all tanks (n = 132) likely due to a Flexibacter infection. There was no difference in weight nor size between fish that died and those that survived (24 g vs. 23.9 g; 11.5 vs. 11.48 cm). At 328 dph, all fish were euthanized using benzocaine (150 mg/L), individually weighted and sexed by in situ gonad examination, or in uncertain cases by microscopic observation of a gonadal squash^76^. From tagging to sexing, fish were kept in identical conditions and were fed ad libitum (BioMar, Nersac, France).

### Parental assignment

A panel of 96 SNP loci extracted from DlabCHIP SNP array design^77^ were submitted to LGC genomics (Middlesex, United Kingdom) for genotyping using the competitive allele-specific PCR (KASP) technology. Fin clips from the 2112 experimental fish and the sires and dams used for the artificial mating were sent to LGC genomics (Hoddesdon, UK) for genomic DNA extraction and genotyping. Parentage assignment was done using the genotyped SNP loci with the R package APIS^78^ with a positive assignment error rate set to 5%.

### Ethical statement

Experiments were performed in accordance with relevant guidelines and regulations provided by the ethic committee (no 36) of the French Ministry of Higher Education, Research and Innovation and the experiment received the following agreement number: APAFIS#19676-2019021915002143. All procedures involving animals were also performed following ARRIVE guidelines (https://arriveguidelines.org/).

### Statistical analysis

Cortisol concentrations in the water were compared using a linear model with multiple predictors: time (days) and treatments as fixed factors (as in Geffroy et al.^69^). We first ran a model with interaction, and then an additive model. The days x treatments interaction was not significant, so that only the output of the additive model was kept. Comparisons of whole-body hormones between treatments were performed with a linear model with a simple predictor (treatments). Since absolute values of quantified hormones were not accurate (according to ISO17025 and EC/657) for fish sampled at 69 dph, we normalized all values to controls for designing figures. The same method was applied for gene expression and monoamines concentration for coherence along the paper (raw values are available in Supplementary material). For gene expression analysis, samples with a geometric mean of the CT for the reference genes above the standard curve point diluted 80 times were removed (this concerned 5 samples: 4 controls and 2 tryptophan). We also detected 3 additional outliers regarding gene expression (one in each treatment) and one outlier (1 tryptophan) following monoamines analysis, using boxplots and a grubbs test. Since all analyses (including sex-ratio) were performed at the group level, these outliers were removed. For gene expression and monoamine concentrations, comparisons were assessed with linear models. To better picture what happened regarding stress in the brain at 132 dph, we combined all monoamines and gene expression data in a Principal Component Analysis (PCA). The difference between groups in individuals’ contributions to the different axis of the PCA was assessed using linear model comparing the three conditions. Differences in sex ratio between treatments were assessed with a generalized linear model with mixed effects (glmer), with a logit link function. The initial tank (during the experimental period, 4 tanks/treatment) of fish was treated as a random factor, while origin (wild/selected) and treatment were fixed factors. Weight differences between treatment, sex and origin at 74, 132, 215 and 328 dph were assessed using a linear model with mixed effects (lmer), with initial tank (during the treatment) as a random factor. Results were considered significantly different when *p* value ≤ 0.05.

## Supplementary Information

Supplementary Information 1.

Supplementary Information 2.
