# Supplementary material for: Parental selection for growth and early-life low stocking density increase the female-to-male ratio in European sea bass
Source: Sci Rep. 2021 Jun 30;11:13620. doi: 10.1038/s41598-021-93116-9 (PMC8245542; doi:10.1038/s41598-021-93116-9)
Supplement: Supplementary file 1 — Supplementary Information 1. [file 41598_2021_93116_MOESM1_ESM.docx]

Supplementary Figure Legend

Figure S1. Ratio between cortisol and other glucocorticoids: 17α-hydroxy-progesterone, 11-deoxycortisol, cortisone and tetrahydrocortisone in fish from the 3 treatments sampled at 34 and 69 dph. Details are provided in the Material and Methods section.
