# Supplementary figures and images for: Parental selection for growth and early-life low stocking density increase the female-to-male ratio in European sea bass
Source: Sci Rep. 2021 Jun 30;11:13620. doi: 10.1038/s41598-021-93116-9 (PMC8245542; doi:10.1038/s41598-021-93116-9)

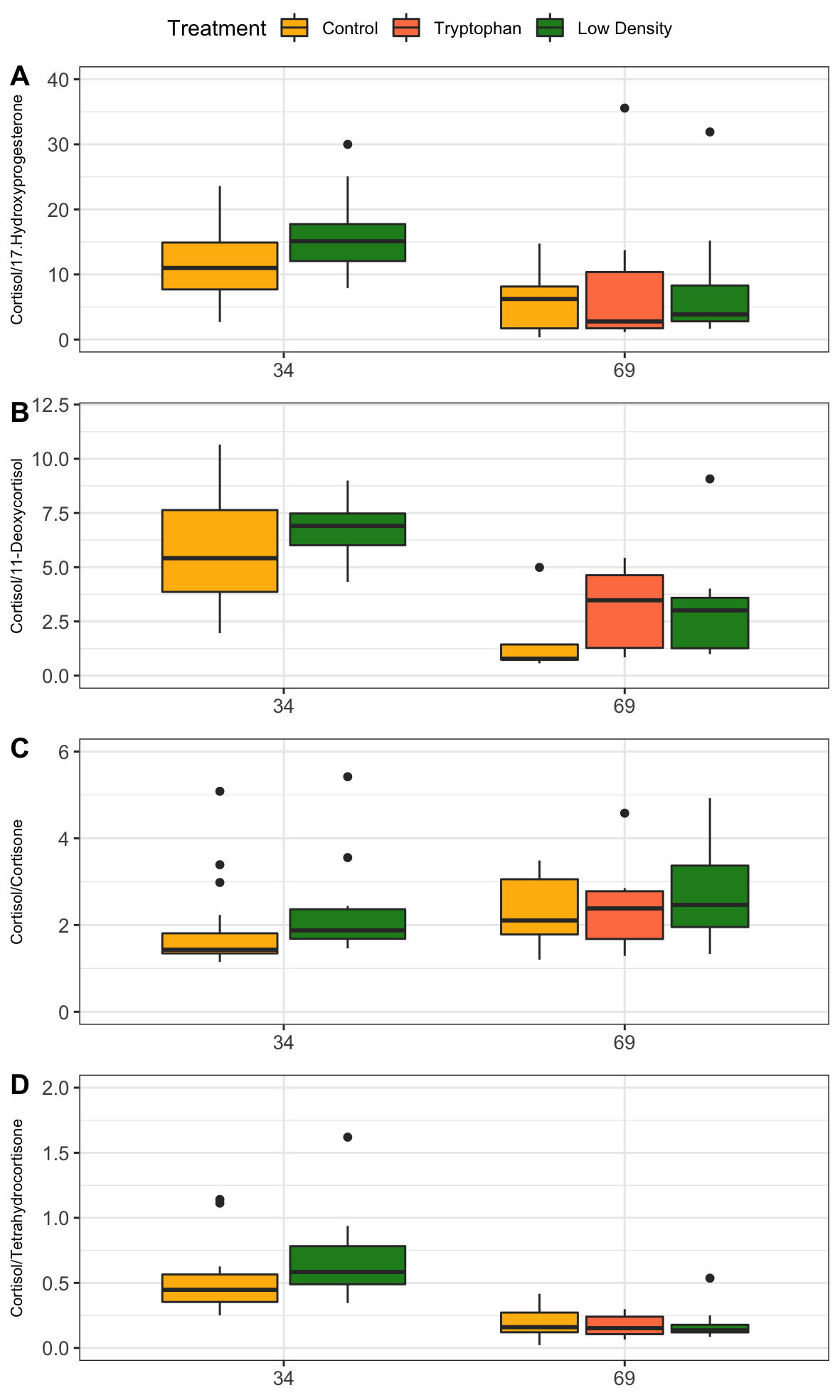

Supplement: Supplementary file 2 — Supplementary Information 2. [file 41598_2021_93116_MOESM2_ESM.tif]
